# Dietary factors and stomach cancer mortality

**DOI:** 10.1038/sj.bjc.6600415

**Published:** 2002-07-15

**Authors:** L T Ngoan, T Mizoue, Y Fujino, N Tokui, T Yoshimura

**Affiliations:** Department of Clinical Epidemiology, Institute of Industrial Ecological Sciences, University of Occupational and Environmental Health, 1-1 Iseigaoka, Yahatanishi-ku, Kitakyushu 807-8555, Japan

**Keywords:** dietary factors, stomach cancer, mortality

## Abstract

The present study examined the relationship between stomach cancer and the low intake of fresh fruit and vegetables and/or a high intake of pickled, preserved or salted foods and frequent use of cooking oil. During 139 390 person–year of follow-up of over 13 000 subjects, 116 died from stomach cancer. Using a Cox proportional hazards–regression analysis of relative risk (RR, 95% CI) controlling for age, sex, smoking and other dietary factors, a significant decline was found with a high consumption of green and yellow vegetables (RR=0.4, 95% CI=0.2–0.9). Reductions of between 40 and 50% were also observed with a high consumption of fresh foods (fruit, cuttle fish, tofu, and potatoes), but these associations were not statistically significant. The risk was significantly increased by the high consumption of processed meat (RR=2.7, 95% CI=1.0-7.4) and by the frequent use of cooking oil (RR=4.0, 95% CI=1.3-11.8). The high consumption of pickled food and traditional soups also increased risk, but not significantly. The findings suggest that a diet high in salt and low in vitamins may be associated with an increase in stomach cancer.

*British Journal of Cancer* (2002) **87**, 37–42. doi:10.1038/sj.bjc.6600415 www.bjcancer.com

© 2002 Cancer Research UK

## 

Stomach cancer was the second most common cause of death from cancer in both sexes in 1990 throughout the world (Pisani *et al*, 1999). In that year, the highest incidence in the world were found in Yamagata, Japan, age standardised incidence rate (ASR) 95.5 and 40.1 per 100 000 in males and females, respectively. These rates were about four times higher than those found in Hawaiian–Japanese (ASR 21.5 and 10.4 per 100 000 in males and females, respectively) (Parkin *et al*, 1997), pointing to the importance of environmental factors. Dietary substances have long been considered to be important risk factors of stomach cancer (Davis, 1989; Nomura, 1996).

Results from previous studies suggested that a diet rich in vitamin C was protective, whereas a diet high in salt might increase the risk of stomach cancer (Mirvish *et al*, 1972; Raineri and Weisburger, 1975; Joossens *et al*, 1996; Zhang *et al*, 2002). Sources of vitamin C include fresh foods, such as green and yellow vegetables, fruit and other fresh foods, while salt ingestion is from processed, pickled, preserved, salted foods, and also seasoning (Kagawa, 1991). Salt is used during processing and preserving, and cooking oil is frequently used in food preparation. The purpose of our present study was to examine the roles in relation to stomach cancer of a diet low in fresh fruit and vegetables or high in pickled, preserved or salted foods and also of frequent use of cooking oil.

## MATERIALS AND METHODS

### Study population

Our cohort study was started in Fukuoka Prefecture in 1986–89, and the follow-up method has been described elsewhere (Fujino *et al*, 2001; Mizoue *et al*, 2000; Utoguchi *et al*, 1997). The number of subjects who were invited to participate and fill in a self-administered questionnaire was 15 417. Among these, 882 subjects did not reply, 1237 did not completely answer all items of this questionnaire, 9 were lost in follow-up, 10 made errors in their follow-up information, and the ages of 29 was unknown. After excluding these subjects, 13 250 (5917 males and 7333 females) aged over 15 had completed the self-administered questionnaire at base line and provided the food frequency details of 25 common food items, remained eligible and were then followed up to 1999. During the 139 390 person–year of follow-up, 116 subjects died from stomach cancer, of which three cases were coded 151.0 (cardia), one case 151.1 (pylorus), two cases 151.2 (pyloric antrum), one case 151.3 (fundus), five cases 151.4 (body of stomach), one case 151.8 (other), and 103 cases 151.9 (unspecified) – (ICD-9). All histological types of stomach cancer cases were analysed together.

### Exposure information

Among 254 items on the self-administered questionnaire, participants were asked to provide their dietary habits regarding the food frequency consumption, such as twice or more per day, once per day, 2–4 times per week, 2–4 times per month, and seldom or never. For subjects recruited in 1986, their frequency of consumption of fresh meat, fish, cuttle fish, tofu, liver, fresh milk, eggs, seaweed, green and yellow vegetables, fruit, potatoes, margarine, use of cooking oil, Japanese miso soup, deep-boiled food, and green tea drinking was obtained. Thereafter, in 1987 and 1989, other food items, such as processed meat and fish, soymilk, milk products, other vegetables, Japanese suimono soup, pickled food, salted food, and sweet cakes, were added to the self-administered questionnaire to collect further dietary information. Among 8161 study subjects recruited in 1987 and 1989, the percentage of subjects who stated that their dietary consumption for these new food items was 93.4–97.4%. Among 13 250 eligible subjects in the whole cohort, the percentage of subjects who stated their dietary consumption of the remaining food items was 91.1–97.3%.

### Statistical analysis

We used the age of subjects in 1987 to categorize age groups as 15–29, 30–39, 40–49, 50–59, 60-69, and 70+. We counted person–years of follow-up for each subject from the starting time of this follow-up until the date of death for 1627 fatal cases; until the date of migration outside the study areas for 1174 subjects; and for 10 449 subjects to the end of last follow-up.

We classified three levels of exposure: low, medium and high, for all food items, except for eggs and other vegetables due to the small number of stomach cancer cases in females at the lowest level (seldom or never). Among these food items, because subjects consumed them at different frequencies, the five levels of food consumption obtained from the self-administered questionnaire were grouped into three levels, according to individual food. For cuttle fish and liver, the low level of exposure was seldom or never, medium was 2–4 times per month, and high was 2–4 times or more per week. For eggs and other vegetables, low exposure level was 2–4 times or less per week and high level was once or more per day (two levels). For fruit, Japanese miso soup, deep-boiled food, and pickled food, the low exposure level was 2–4 times or less per week, medium was once per day, and high was twice or more per day. For the remaining food items, low exposure level was 2–4 times or less per month, medium was 2–4 times per week, and high was once or more per day (Tables 2

**Table 2:**
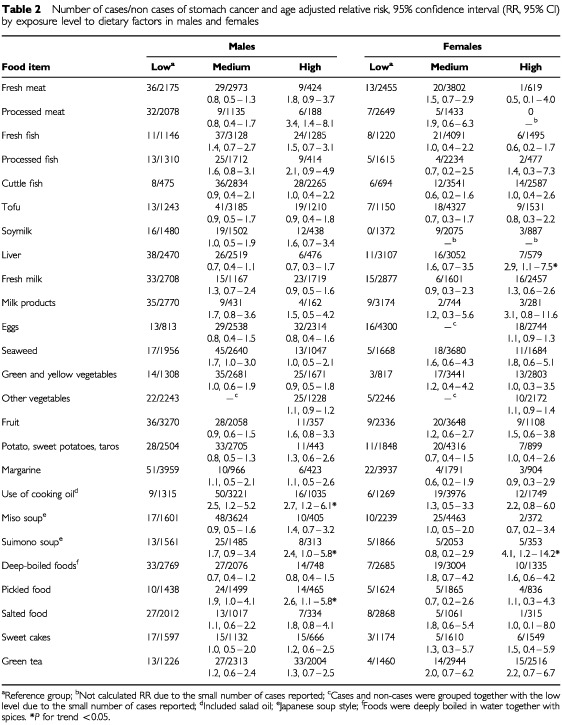
Number of cases/non cases of stomach cancer and age adjusted relative risk, 95% confidence interval (RR, 95% CI) by exposure level to dietary factors in males and females

and 3

**Table 3:**
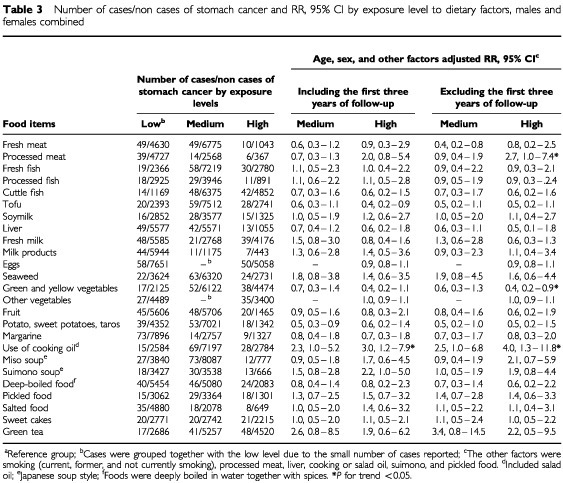
Number of cases/non cases of stomach cancer and RR, 95% CI by exposure level to dietary factors, males and females combined

).

A Cox proportional hazards–regression analysis was used to estimate relative risk and 95% confidence interval (RR and 95% CI) (STATA, 1999). The other factors are tobacco smoking (current, former, and currently not smoking), alcohol (current or currently not drinking), history of chronic gastric symptoms (yes or no), occupation (permanent or nonpermanent work), and coffee drinking (daily or occasionally). These factors control possible confounding factors (Haenszel *et al*, 1972; Hoey *et al*, 1981; Hisashige *et al*, 1983; Jeyaratnam *et al*, 1987; Kono *et al*, 1988; Yin *et al*, 1989; Hirayama, 1990; Kneller *et al*, 1990; Une *et al*, 1995; You *et al*, 1995; Galanis *et al*, 1998).

Stomach cancer patients who died during the first years of the follow-up might have changed their dietary habits at the time of registration. Therefore, the data was analysed, both including and excluding the first three years of follow-up.

## RESULTS

Characteristics of the study subjects at base line are presented in Table 1

**Table 1:**
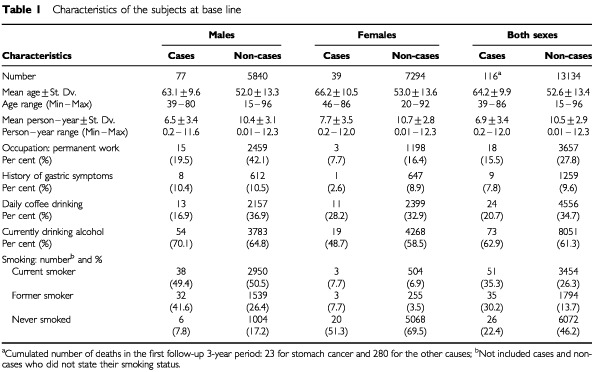
Characteristics of the subjects at base line

. A total of 116 cases of deaths from stomach cancer were identified, 77 males and 39 females. The mean age of non-stomach cancer patients when compared to stomach cancer patients was younger by more than 10 years in both sexes. There was no significant association with stomach cancer and gastric symptoms (RR=1.2, 95% CI=0.6–2.4), coffee drinking (RR=1.0, 95% CI=0.9–1.1), alcohol consumption (RR=1.0, 95% CI=0.9–1.1), or occupation (RR=1.0, 95% CI=0.9–1.1) in this study population, so these factors were excluded from the final model. The high consumption of green and yellow vegetables was similar in cases (35.5%) and non-cases (35.2%), although among cases, it was higher (47.6%) in the first six years of follow-up than in the later period (27.7%). A greater proportion of subjects with a high fruit consumption was found among cases (17.7%) than non-cases (11.5%), and also in the first six years of follow-up (20.6%) than in the later period (16.2) among cases.

Age adjusted RR, 95% CI by gender is presented for individual foods in Table 2. A high consumption of processed meat was associated with a significantly increased the risk of stomach cancer in males (RR=3.4, 95% CI=1.4–8.1, *P* for trend=0.07) but not in females (RR=1.9, 95% CI=0.6–6.3). An increased RR was seen with the frequent use of cooking oil (2.7, 95% CI=1.2–6.1, *P* for trend <0.05 in males and 2.2, 95% CI=0.8–6.0, *P* for trend=0.08 in females). A high consumption of pickled foods was associated with a significantly increased risk of stomach cancer in males (RR=2.6, 95% CI=1.1–5.8, *P* for trend <0.05) but not in females (RR=1.1, 95% CI=0.3–4.3). A high consumption of suimono soup significantly increased the risk of stomach cancer in both males (RR=2.4, 95% CI=1.0–5.8, *P* for trend <0.05) and females (RR=4.1, 95% CI=1.2–14.2, *P* for trend <0.05). Data for males and females combined and controlling for age and sex showed that the high consumption of green and yellow vegetables, tofu, and cuttle fish slightly decreased the risk of stomach cancer (RR=0.9, 95% CI=0.5–1.6, RR=0.9, 95% CI=0.5–1.6, and RR=0.9, 95% CI=0.5–1.8, respectively). The increased risk of stomach cancer associated with the high consumption of processed meat, suimono soup, and the frequent use of cooking oil remained.

Table 3 presents the findings adjusted for sex, age, smoking, and other dietary factors (processed meat, liver, cooking oil, suimono, and pickled food), both including and excluding the first three-year of follow-up. A significant decline in the risk of stomach cancer was found with a high consumption of green and yellow vegetables (RR=0.4, 95% CI=0.2–0.9, *P* for trend <0.05). Reductions of between 40 and 50% were also observed for the high consumption of fruit, cuttle fish, tofu, and potatoes, but these associations were not statistically significant. In contrast, the risk of stomach cancer was significantly increased with the high consumption of processed meat (RR=2.7, 95% CI=1.0–7.4, *P* for trend <0.05) and the frequent use of cooking oil (RR=4.0, 95% CI=1.3–11.8, *P* for trend <0.05). Elevations in stomach cancer risk were also observed with a high consumption of pickled food (RR=1.4, 95% CI=0.6–3.3), miso soup (RR=2.1, 95% CI=0.7–5.9), and suimono soup (RR=1.9, 95% CI=0.8–4.4), but again these were not statistically significant.

## DISCUSSION

Our study subjects were recruited from the Fukuoka general population, and a large number of subjects provided their frequency of food consumption for 25 common food items. The frequency of food consumption and other factors were recorded before diagnosis of stomach cancer and other diseases, thus avoiding recall bias. These several methodological advantages helped us to evaluate the association between individual dietary items and stomach cancer. Our cohort study showed a beneficial effect of green and yellow vegetables and the risk of processed meat and the frequent use of cooking oil. However, despite the moderately large number of cases, there was insufficient power to detect a statistically significant association for many foods, and there were small numbers in most exposure categories of interest. The small number of cases, especially in females, may be responsible for the inconsistencies and a conflicting observation (as in liver and deep-boiled food) between males and females (Table 2). Nevertheless, as fresh foods are rich in vitamins and low in salt and Japanese traditional foods are poor in vitamins and rich in salt, the data are suggestive that a diet high in salt and low in vitamins may be associated with stomach cancer.

Green and yellow vegetables are very rich in vitamin C, and has been found to be protective against stomach cancer in a number of experimental and epidemiological studies. Thus, vitamin C may inhibit gastric cancer cell growth, be a possible means of blocking the formation of human carcinogenic N-Nitroso compounds, and may reduce nitrite concentration by 43% (Mirvish *et al*, 1972; Raineri and Weisburger, 1975; Zhang *et al*, 2002). A prospective cohort study found a significantly decreased risk of progression to dysplasia or gastric cancer among subjects with high blood ascorbic acid level (OR=0.2, 95% CI=0.1–0.7) (You *et al*, 2000). A similar observation was seen in a case–control study (OR=0.37, 95% CI=0.16–0.86) (Boeing *et al*, 1991). A high consumption of vegetables was associated with a significantly reduced risk of stomach cancer (RR ranked from 0.38–0.40 and RR=0.5, 95% CI=0.3–0.9) (Haenszel *et al*, 1972; Ahn, 1997), as was a high consumption of vegetables and fruit (You *et al*, 1988; Nomura *et al*, 1995; Galanis *et al*, 1998; Ji *et al*, 1998; Terry *et al*, 1998). These findings were consistent with our results for green and yellow vegetables (RR=0.4, 95% CI=0.2–0.9, *P* for trend <0.05) (Table 3).

Kagawa (1991) confirmed that Japanese preserved foods, such as processed meat, pickled vegetables, and traditional noodles or soup, are rich in sodium and low or null in vitamin C. High salt foods were associated with an increased risk of stomach cancer (Haenszel *et al*, 1972; Tuyns, 1988; Nazario *et al*, 1993; Ramon *et al*, 1993; Lee *et al*, 1995; Ahn, 1997; Huang *et al*, 2000). These earlier results are also in general agreement with our finding for high consumption of processed meat (RR=2.7, 95% CI=1.0–7.4, *P* for trend <0.05).

A high frequent use of cooking oil significantly increased the risk of stomach cancer in both males and females (Tables 2 and 3). Deep-oil-fried foods are common traditional Japanese foods (Tsugane *et al*, 2001; Ogawa *et al*, 2002) and produce human carcinogens at high cooking oil temperatures. Cooking oil fumes contain high concentrations of human carcinogens, such as BaP and DBahA, and heterocyclic aromatic amines, due to high frying temperatures (Li *et al*, 1994; Sinha *et al*, 1994; Skog *et al*, 1995). Benzo(a)pyren, chrysene, and dibenzathracene have been detected at significant levels in oil fried vegetables and fish (Sivaswamy *et al*, 1991). Frying also leads to the formation of both the aminomethylimidazoquinoline and the carboline types of HAs (Chiu *et al*, 1998). Another prospective cohort study found that a high consumption of deep-fried food increases the risk of stomach cancer (RR=1.71, 95% CI=0.67–4.34) (Kato *et al*, 1992), which was also observed among subjects who consumed fried foods frequently (OR=2.3, 95% CI=1.6–3.2) (Ji *et al*, 1998).

Changes of drinking and eating habits due to ill health among stomach cancer patients have been well addressed (Jacobsen *et al*, 1986). A small increase in the risk of stomach cancer among cases exposed to the high consumption of fruit in both males and females before controlling for other dietary factors might be due to changes of drinking and eating habits causing an ill condition among stomach cancer patients (Table 2). After adjustment for sex, age, smoking, and other dietary factors (processed meat, liver, cooking oil, suimono, and pickled food), a slight protective effect of fruit consumption against developing stomach cancer was observed, both including and excluding the first three-year period of follow-up (Table 3).

Our study had certain limitations. We did not obtain information at base line on *Helicobacter pylori* infection (*HP*). Among Japanese, HP infection significantly increases the risk of stomach cancer (Blaser *et al*, 1993; Fukuda *et al*, 1995; Kikuchi *et al*, 1995; Watanabe *et al*, 1997). However, a previous prospective cohort study showed that *HP* infection significantly increased the risk of stomach cancer in males (RR=2.59, 95% CI=1.03–6.50) but not in females (RR=0.99, 95% CI=0.36–2.68) (Yamagata *et al*, 2000). A high consumption of pickled vegetables and miso soup significantly increased *HP* infection (OR=1.9, 95% CI=1.10–3.30 and OR=1.60, 95% CI=1.03–2.49, respectively) (Tsugane *et al*, 1994). High-salt diets contribute to expansion of *HP* colonisation (Fox *et al*, 1999), therefore, our present results may be affected by *HP* infection. Another limitation was that the self-administered questionnaire had not been validated. Many other traditional Japanese foods/recipes that have been reported to be risk factors of stomach cancer (Mochi-glutinous rice cake: OR ranked from 1.03–1.80, traditional Japanese style salad: RR=3.10, 95% CI=1.40–6.85) were not included in the present study (Haenszel *et al*, 1976; Kato *et al*, 1992). We believe that our results would have been better if these factors had been considered for controlling possible confounding factors in the analysis models.
